# Transgenerational genomic analyses reveal allelic oscillation and purifying selection in a gut parasite *Nosema ceranae*

**DOI:** 10.3389/fmicb.2022.927892

**Published:** 2022-11-01

**Authors:** Xiuxiu Wei, Jialan Zheng, Jay D. Evans, Qiang Huang

**Affiliations:** ^1^Jiangxi Key Laboratory of Honeybee Biology and Beekeeping, Jiangxi Agricultural University, Nanchang, China; ^2^Honeybee Research Institute, Jiangxi Agricultural University, Nanchang, China; ^3^College of Forestry, Jiangxi Agricultural University, Nanchang, China; ^4^USDA-ARS Bee Research Laboratory, Beltsville, MD, United States

**Keywords:** microsporidian, SNV, selection, honey bee, mutation

## Abstract

Standing genetic variation is the predominant source acted on by selection. Organisms with high genetic diversity generally show faster responses toward environmental change. *Nosema ceranae* is a microsporidian parasite of honey bees, infecting midgut epithelial cells. High genetic diversity has been found in this parasite, but the mechanism for the parasite to maintain this diversity remains unclear. This study involved continuous inoculation of *N. ceranae* to honey bees. We found that the parasites slowly increased genetic diversity over three continuous inoculations. The number of lost single nucleotide variants (SNVs) was balanced with novel SNVs, which were mainly embedded in coding regions. Classic allele frequency oscillation was found at the regional level along the genome, and the associated genes were enriched in apoptosis regulation and ATP binding. The ratio of synonymous and non-synonymous substitution suggests a purifying selection, and our results provide novel insights into the evolutionary dynamics in microsporidian parasites.

## Introduction

Microsporidia comprise a large group of obligate intracellular parasites, which forms an early branch in the fungal kingdom (Capella-Gutiérrez et al., 2012; Choi and Kim, 2017). Microsporidian genomes were generally compact, ranging between 2 and 5 Mbp (Ndikumana et al., 2017), arguably because they rely on host cells for much of their metabolic processes. Interestingly, one exceptionally large genome of 51 Mbp has been reported, and this species maintains transportable elements, which are exceedingly rare in microsporidia generally (Desjardins et al., 2015). Conventional mitochondria are lost during evolution, leaving tiny mitochondrially derived organelles called mitosomes (Burri et al., 2006). Microsporidian infections have been reported to inhibit host apoptosis, suppress host immune responses, and enhance host metabolism (Scanlon et al., 1999; del Aguila et al., 2006; Antunez et al., 2009; Cuomo et al., 2012; Higes et al., 2013; Martín-Hernández et al., 2017). More recently, a parasitic miRNA was found to be a virulence factor for one microsporidian to establish infection in silkworms (Dong et al., 2021).

*Nosema ceranae* is a microsporidian parasite that infects epithelial cells of honey bee midgut tissue (Higes et al., 2007). *N. ceranae* was first described in the Asian honey bee *Apis cerana*, which has established infection in the European honey bee *Apis mellifera* globally (Fries et al., 1996; Higes et al., 2006; Huang et al., 2007). The genome size of *N. ceranae* is 8.8 Mbps, with 2,280 protein-coding genes (Huang et al., 2021). The infection starts with the ingestion of *N. ceranae* spore-contaminated nectar. A parasite proliferation cycle lasts approximately 4 days, defined by life stages from meronts and sporonts to mature spores (Higes et al., 2007). Infected cells eventually burst to release a large number of spores. Parasites can then germinate to infect neighboring healthy epithelial cells or can be expelled into the environment through feces. Honey bees infected by *N. ceranae* showed reduced life span, enhanced energetic burden, and impaired navigation and memory (Antunez et al., 2009; Mayack and Naug, 2009; Eiri et al., 2015; Gage et al., 2018). In extreme conditions, the infection may lead to colony collapse (Higes et al., 2008; Botías et al., 2013). Using a set of genetic markers, high genetic diversity of *N. ceranae* has been found. Surprisingly, this genetic diversity can be even higher within a colony than among colonies (Gómez-Moracho et al., 2014, 2015). Additionally, the genetic variation of *N. ceranae* was minor among geographically distant locations (Pelin et al., 2015). The host shapes the parasite diversity, and the parasite drives the host diversification (Kamiya et al., 2014; Betts et al., 2018; McNew et al., 2021). It remains unclear how *N. ceranae* could maintain high levels of polymorphism. We hypothesized that mutation and frequency-dependent selection together shape the adaptation of the parasite. To validate the above hypothesis, the single nucleotide variants (SNVs) of *N. ceranae* were quantified over three successive generations to assess the frequency of novel and lost SNVs, and allele oscillation from parental to offspring spores.

## Materials and methods

### Parasite isolation, inoculation, and sample collection

To harvest sufficient spores, 300 honey bee foragers were randomly collected from five heavily infected honey bee (*A. mellifera*) colonies in the experimental apiary of Jiangxi Agricultural University following a regular survey. Midguts of these honey bees were dissected and homogenized to isolate *N. ceranae* spores *via* centrifugation (Fries et al., 2013). Then, the spores were purified using Percoll gradient centrifugation and confirmed as pure *N. ceranae* by species-specific fragment size of a conventional PCR (Chen et al., 2013; Fries et al., 2013). The purified spores were defined as F0_spores. A subset of these F0_spores was used to extract genomic DNA using cetyltrimethylammonium bromide (CTAB) (Chen et al., 2013). The remaining F0_spores were used to inoculate newly emerged honey bees. Sealed honey bee (*A. mellifera*) brood frames were kept in an incubator (34 ± 1°C, 60% relative humidity). In total, 200 newly emerged honey bee workers were individually fed with 2 μl sucrose solution containing 10^5^ *N. ceranae* F0 spores.

Additionally, 200 newly emerged honey bee workers were fed with 2 μl sucrose solution as the F1 uninfected group. During the experiment, cohorts were divided into 4 cups containing 50 bees each and maintained on 50% sucrose solution *ad libitum* in the incubator. To harvest sufficient spores to infect a new batch of honey bees, the midgut was dissected from individual honey bees at 14 days postinfection (dpi), and F1 spores were collected. A subset of F1 spores was again used to extract genomic DNA, and the remaining F1 spores were used to inoculate 100 newly emerged honey bees due to a limited number of spores. In total, 100 newly emerged honey bee workers were fed with 2 μl sucrose solution as the F2 uninfected group. At 14 dpi, the midgut was dissected from individual honey bees and then pooled for spore purification, defined as F2 spores. The experiment was duplicated. Overall, two libraries for F0_spores, two libraries for F1 spores, and two libraries for F2 spores were prepared and sequenced on Illumina Hiseq 2000 platform in Novogene Co.

### Bioinformatic and statistics

The quality of DNA sequencing reads was processed with Fastp and validated with FastQC (Chen et al., 2018). DNA sequencing reads were aligned to the *N. ceranae* genome (Ncer 3.0, GCA_004919615.1) using BWA with default parameters (Li and Durbin, 2009; Huang et al., 2021). The SNVs were identified and annotated using the Picard-GATK-SNPEFF pipeline (Van der Auwera et al., 2013). The SNVs with a quality score of less than 50 and locus alignment coverage of less than 10 were removed. Enrichment GO terms were analyzed using TopGO, R (Alexa and Rahnenfuhrer, 2021). The SNVs among F0, F1, and F2 spores were analyzed using chi-squared tests implemented using R (R Core Team, 2013). The lost and novel SNVs among the two generations were analyzed using paired *t*-test, implemented using R (R Core Team, 2013). The ratio of π*_*non–synonymous*_*/π*_*synonymous*_* (π*_*a*_/*π*_*s*_*) was calculated using SNPGenie (Nelson et al., 2015). The genome diversity π, *Watterson’s* θ, and corrected *Tajima’s D* were calculated using Popoolation (Kofler et al., 2011a). The fixation index *F*_*ST*_ was calculated using Popoolation2 (Kofler et al., 2011b). The recombination rate was calculated based on the allele frequency using ReLERNN (Adrion et al., 2020). DNA sequencing reads have been deposited in NCBI under BioProject PRJNA784016.

## Results

### The number of novel and lost single nucleotide variants were balanced between parental and offspring spores

**FIGURE 1:**
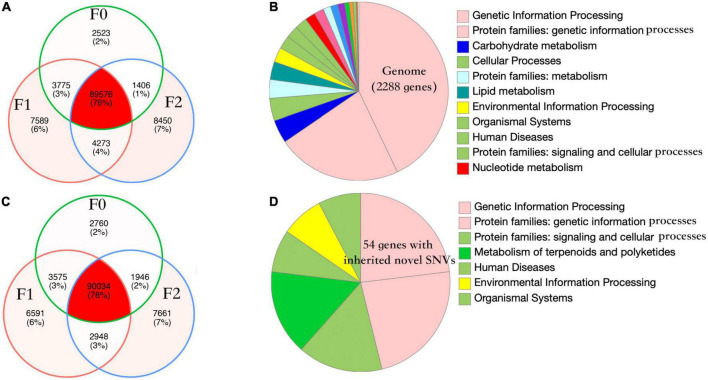
Number of single nucleotide variants (SNVs) and the gene function of *Nosema ceranae*. Venn diagram of parasite SNVs isolated from the three generations in two replicates **(A,C)**. The number of lost SNVs was balanced with the number of novel SNVs, which could be the mechanism for the parasite to maintain high polymorphism. The pattern of SNVs was highly congruent between the two replicates (Pearson’s chi-squared test, *P* > 0.05). The function of protein-coding genes in the parasite genome **(B,D)** and the function of 54 genes with novel non-synonymous SNVs. The genes were enriched in metabolism compared with the genome (Fisher’s exact test, *P* < 0.001).

**FIGURE 2:**
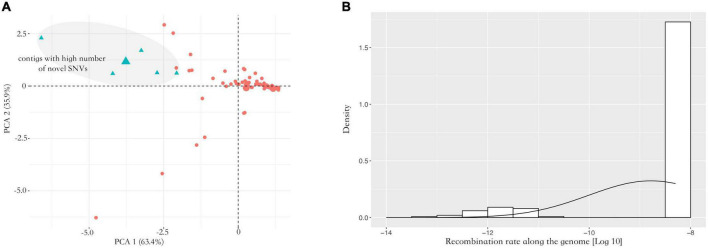
Analysis of allele frequency. **(A)** PCA analysis of the novel single nucleotide variants (SNVs) along the genome. The contigs showed a significant difference in generating novel alleles. The contigs highlighted with blue showed a higher tendency to generate novel SNVs across two replicates (one sample *t*-test, adjusted *P* < 0.05). **(B)** Inferred recombination rate along the genome. Overall, the inferred recombination rate was small along the genome with 5.14e-9 crossover per bp. The X-axis indicates the recombination rate after the log10 transformation. The Y-axis indicates density.

*Nosema ceranae* spores were not found in the uninfected groups. For the spores purified from infected honey bees, 104 ± 7 million reads (mean ± SD) were aligned to the *N. ceranae* genome (150 bp paired reads, over 1,000 genome coverage), which accounted for 93.2% of all sequenced reads. In F0 spores, 97,279 and 98,314 SNVs were identified in two replicates with 96.8% overlap, reflecting approximately 11 SNVs per Kbp (Figure 1). Additionally, over 95% SNVs were heterozygous. Within coding regions, 7,352 non-synonymous and 8,695 synonymous SNVs were identified in F0 spores. From F0 to F1 spores, 103 synonymous and 200 non-synonymous SNVs were lost. Comparatively, 314 synonymous and 350 non-synonymous novel SNVs were gained in F1 spores. From F1 to F2 spores, 302 synonymous and 314 non-synonymous SNVs were lost. Meanwhile, 982 synonymous and 1,228 non-synonymous novel SNVs were gained. The dynamic of non-synonymous SNVs was significantly stronger than synonymous SNVs from F0 to F2 spores (paired *t*-test, *P* < 0.01). Among the novel non-synonymous SNVs in F1 spores, 198 SNVs were maintained in F2 spores located within 54 genes. Using the genome as the background, the 54 genes were significantly enriched in the metabolism pathway (Fisher’s exact test, *P* < 0.001). After normalizing the contig length, the contig SMUP01000049.1 (13.1 novel SNVs per 10 kbp), SMUP01000091.1 (8.6 novel SNVs per 10 kbp), SMUP01000014.1 (8.0 novel SNVs per 10 kbp), SMUP01000071.1 (6.7 novel SNVs per 10 kbp), and SMUP01000003.1 (6.2 novel SNVs per 10 kbp) showed a significantly higher tendency to generate novel SNVs compared with the genome (one sample *t*-test, adjusted *P* < 0.05) (Figure 2A). There were 269 genes embedded in the above contigs, which were enriched in the cell cycle (GO: 0010971, adjusted *P* < 0.05) and ATP binding (GO: 0005524, adjusted *P* < 0.001).

### Genome recombination and allele frequency oscillation from parental to offspring spores

**FIGURE 3:**
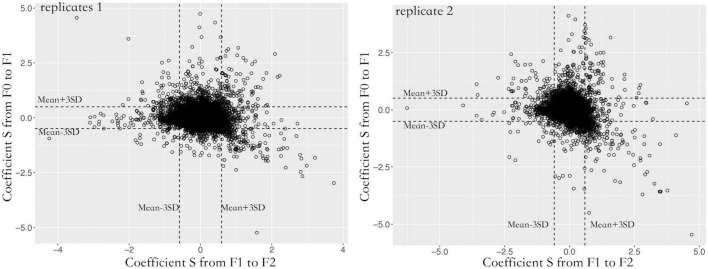
Allele frequency oscillation over three generations in two replicates. For each generation, the coefficient *S* significantly deviated from random, suggesting the existence of oscillation. To reduce the false positive calling, only the loci that fell within 99.9% confidence were considered under oscillation. The X-axis indicates allele frequency variation (coefficient *S*) from F1 to F2 spores. The Y-axis indicates allele frequency variation (coefficient *S*) from F0 to F1 spores.

A total of 87,129 SNVs were inherited from F0 to F2 spores in both replicates. Over 95% of loci were heterozygous with an average alternative allele frequency of 0.38. Contigs with lower recombination rates were rarely found. Most contigs were homogeneous with 5.14e-9 cross-over per bp (Figure 2B). To quantify the allele oscillation, the allele frequency variation was calculated between parents and offspring, denoted as coefficient *S*. When offspring inherits the same allele frequency from their parents, the coefficient *S* equals 0. In our data, the coefficient *S* significantly deviated from F0 to F1, as well as from F1 to F2 in both replicates (one sample *t*-test, adjusted *P* < 0.001) (Figure 3). By comparing with the genome, the genes associated with the selected loci were significantly enriched in negative regulation of apoptosis (GO: 0043066, adjusted *P* < 0.05) and ATP binding (GO: 0005524, adjusted *P* < 0.001).

### Genetic differentiation accumulated over generations

Genome diversity (π) slowly increased from F0 (0.0084 ± 0.0001) and F1 (0.0087 ± 0.0001) to F2 (0.0088 ± 0.0001). *Tajima’s D* was positive, and the ratio of π*_*a*_/*π*_*s*_* was less than 1 for the parasites in all three generations. The fixation index *F*_*ST*_ was 0.0015 ± 0.0001 between F0 and F1 spores, which increased by twofold (0.0042 ± 0.0001) between F1 and F2 spores. After two parasite generations, the index *F*_*ST*_ increased by fourfold from F0 to F2 spores (Table 1).

**TABLE 1:** Population genetic statistics for the three generations of *Nosema ceranae* spores (mean ± SE).

*Parasites*	π *_*a*_/*π *_*s*_*	π	*Waterson’s* θ	*Tajima’s D*	F0 (*F*_*ST*_)	F1 (*F*_*ST*_)
F0	0.2027 ± 0.0122	0.0084 ± 0.0001	0.0080 ± 0.0001	0.7980 ± 0.0159		
F1	0.2009 ± 0.0107	0.0087 ± 0.0001	0.0088 ± 0.0001	0.3367 ± 0.0152	0.0015 ± 0.0001	
F2	0.2034 ± 0.0117	0.0088 ± 0.0001	0.0085 ± 0.0001	0.7194 ± 0.0157	0.0071 ± 0.0001	0.0042 ± 0.0001

π_a_ indicates the diversity at non-synonymous sites, and π_s_ indicates the diversity at synonymous sites.

The fixation index F_ST_ of the parasites in three generations was pairwise analyzed, which increased substantially with each generation.

## Discussion

Host-parasite coevolution is a process of reciprocal allelic oscillation between antagonist species and is often characterized by the Red Queen Hypothesis, which is a central theorem in coevolutionary biology (Liow et al., 2011; Brockhurst et al., 2014; Rabajante et al., 2016; Martínez-López et al., 2021). High-standing genetic variation allows fast responses to environmental change (Lai et al., 2019). *N. ceranae* is a native parasite of Eastern honey bees (*A. cerana*), which has successfully established widespread infections in Western honey bees (*A. mellifera*). The parasite showed high virulence in the novel host, and infected honey bees showed a shortened life span (Eiri et al., 2015), suppressed apoptosis (Antunez et al., 2009; Kurze et al., 2015; Martín-Hernández et al., 2017), and arguably are prone to colony collapses (Higes et al., 2008; Botías et al., 2013). Using a set of genetic markers, the diversity of this parasite was found higher within a honey bee colony than among colonies (Gómez-Moracho et al., 2014, 2015). In our data, the number of identified SNVs was congruent with a previous study (Ke et al., 2021). The numbers of inherited, lost, and novel SNVs were highly congruent between the two replicates, suggesting that selection is not random at the colony level. Additionally, we found minor variance between the replicates, suggesting that genetic diversity is higher within a colony than among colonies, as indicated previously (Gómez-Moracho et al., 2014; Pelin et al., 2015). Previously, the recombination event was reported in *N. ceranae* (Pelin et al., 2015). In our study, the calculated recombination rate was orders of magnitude smaller than those of other fungal pathogens (Heinzelmann et al., 2020; Wyka et al., 2022). Inhibiting apoptosis and ATP acquisition were essential to the success of *N. ceranae* infection. When apoptosis was facilitated, infected honey bees were tolerant of the infection (Kurze et al., 2015). Suppressing the expression of ATP transporters also reduced *N. ceranae* proliferation (Paldi et al., 2010). In our data, the alleles associated with apoptosis and ATP binding were under constant oscillation, which supports the antagonistic allele oscillation under the Red Queen hypothesis (Papkou et al., 2019). Anthropocene could also shape the genome selection of the parasite (Ptaszyńska et al., 2021), as well as the foraging behavior of honey bees. For example, parasites can quickly disperse through commonly visited flowers, which may decrease colony level variance (Graystock et al., 2015). The transporting pollination activities may further enhance the parasite dispersal at a larger scale (Proesmans et al., 2021). Furthermore, plant diversity can strongly impact the honey bee diet quality, which may shape the susceptibility to pathogens (Ptaszyńska et al., 2021). Genetic drift and selection decrease genetic variation, while mutation, gene flow, and horizontal gene transfer increase genetic variation (Schulte et al., 2013; Ellegren and Galtier, 2016). In our data, a strong fraction of SNVs was lost during the bottleneck caused by a single inoculation. Meanwhile, mutations led to ample novel SNVs. As the parasite inoculation was employed in lab conditions, the impacts of gene flow were minimal. This suggests that a balance between lost and novel SNVs maintains the high genetic diversity in *N. ceranae*. In our data, the accumulating π suggests a slow accumulation of genetic diversity over generations. Genome diversity π was slightly higher than *Watterson’s* θ, leading to a small positive *Tajima’s D*, which suggests a small number of low-frequency alleles (Fu and Li, 1993; Stajich and Hahn, 2005). The ratio of π*_*a*_/*π*_*s*_* was less than 1 in all three parasite generations, which suggests that the parasite genome is going through purifying selection as found in historical samples (Huang et al., 2016). The fixation index increased fourfold from F0 to F2 spores, suggesting a genome diversification (Jackson et al., 2015). The parasite can transmit among bee species, which might have posed additional selective pressure on the parasite to adapt to multiple hosts. In yeast, static environments favor small numbers of fit genotypes, dramatically decreasing genetic diversity. In contrast, fluctuating environments enrich genotypes and partially contribute to the maintenance of genetic diversity through balancing selection (Abdul-Rahman et al., 2021). Additionally, parasites show elevated diversity after infecting diverse host populations compared with monocultures (Ekroth et al., 2021), suggesting that host genotype shapes parasite diversity. To adapt to a highly polyandrous honey bees, *Nosema* parasites may be under the pressure to maintain high polymorphism. In Denmark, honey bees tolerant toward *N. ceranae* infection showed positive selection (Huang et al., 2014).

*N. ceranae* is a notorious parasite that weakens honey bee health. In this study, we partially explained the mechanism underlying this parasite’s observed high genetic diversity. We need to point out that multiple proliferation cycles may have occurred for each sampling generation because the infection period was three times more than a typical parasite life cycle. We found that a balance between the novel and lost SNVs drives the maintenance of genetic diversity in this species. Additionally, the allele frequencies of genes essential for parasite infection showed oscillation, supporting the Red Queen hypothesis. Our study enhances the understanding of the genetic structure, facilitated by the global spread of this parasite while providing a blueprint for parasite control.

## Data availability statement

The datasets presented in this study can be found in online repositories. The names of the repository/repositories and accession number(s) can be found below: https://www.ncbi.nlm.nih.gov/, PRJNA784016.

## Author contributions

XW conducted the experiment. QH designed the experiment. XW, JZ, JE, and QH organized the manuscript. All authors contributed to the article and approved the submitted version.
